# Administrator Turnover: The Roles of District Support, Safety, Anxiety, and Violence from Students

**DOI:** 10.3390/bs14111089

**Published:** 2024-11-13

**Authors:** Andrew H. Perry, Linda A. Reddy, Andrew Martinez, Susan D. McMahon, Eric M. Anderman, Ron A. Astor, Dorothy L. Espelage, Frank C. Worrell, Taylor Swenski, Kailyn Bare, Christopher M. Dudek, Jared Hunt, Adriana I. Martinez Calvit, Hyun Ji Lee, Xi Liu

**Affiliations:** 1 Department of Educational Studies, The Ohio State University, Columbus, OH 43210, USA; 2 Graduate School of Applied and Professional Psychology, Rutgers University, New Brunswick, NJ 08901, USA; 3 New York Center for Justice Innovation, New York, NY 10018, USA; 4 Department of Psychology, DePaul University, Chicago, IL 60614, USA; 5 Social Welfare and Education, University of California-Los Angeles, Los Angeles, CA 90095, USA; 6 School of Education, University of North Carolina at Chapel Hill, Chapel Hill, NC 27599, USA; 7 Berkeley School of Education, University of California-Berkeley, Berkeley, CA 94720, USA; 8 Department of Psychology, North Carolina State University, Raleigh, NC 27695, USA

**Keywords:** administrator turnover, administrator wellbeing, district-level support, violence against administrators

## Abstract

Researchers have examined the importance of school administrative support for teacher safety, victimization, anxiety, and retention; however, studies to date have rarely focused on school administrators’ perceptions of support by their district leaders, and its relation to administrators’ anxiety/stress, safety, and their intentions to transfer or quit their jobs. In the current study of 457 PreK-12th grade school administrators in the United States, structural equation modeling was used to examine relations between administrators’ perceptions of support from their district leaders and their anxiety/stress, safety, and intentions to transfer or quit their jobs. Administrator experiences of violence by student offenders served as a moderator. Results indicated that administrators’ perceptions of district leaders’ support were associated with lower intentions to transfer or quit their positions both directly and indirectly as a function of decreased anxiety/stress. District support was positively related to administrator safety, particularly for administrators who reported experiencing more student violence. Findings highlight the importance of district support of administrators for reducing mental health concerns and transfer/quit intentions in the context of student violence against school administrators. Implications of findings for research and practice are presented.

## 1. Introduction

School administrators play a pivotal role as leaders who are responsible for fostering a positive school climate and educator and student success [1,2]. Accordingly, turnover among school administrators can disrupt school functioning. An examination of U.S. public school principals during the 2021–2022 school year by the National Center for Education Statistics [3] found that approximately 17% of principals either transferred schools or quit their jobs, with principals employed at schools in impoverished communities being most likely to leave their jobs compared to principals employed at schools in affluent communities. Despite school administrators’ vital roles in school systems, most research on turnover in K-12 schools has focused on teachers, with findings projecting a serious short-term teacher shortage [4,5]. Similar to teachers, there is preliminary evidence of an impending shortage of school administrators, with one report suggesting that as many as one in two school leaders are considering leaving their profession due to overwhelming stress levels [6]. However, administrator turnover, especially as a consequence of administrator wellbeing, has received limited attention within the scientific literature.

School administrator turnover can have a myriad of consequences on the school community, including negative effects on school climate [7], turnover among teaching staff [8], and student achievement [9]. The pivotal role that administrators play in their school systems further explains why administrators and principals might be inclined to leave. Administrators are often responsible for implementing and monitoring school policies and practices and are accountable for their success. However, failed policies and practices can be exceedingly frustrating for administrators, as they are often not the architects of federal and/or state policies and practices [10].

Overall, understanding the antecedents that lead to school administrators transferring schools or quitting their jobs is of crucial importance. In this study, we propose that administrator wellbeing is a primary mechanism through which job-related stressors contribute to intentions to transfer or quit. Additionally, we propose that support from district leaders may help to mitigate the strain on administrator wellbeing, which should then relate to decreases in attrition among school administrators.

### 1.1. Antecedents to Administrator Turnover

Several reasons for administrator turnover have been postulated, including the demands of the job and minimal resources [11]. For example, over 10% of high school administrators report that having more teachers and staff available would decrease their likelihood of leaving the profession [6]. Additionally, having more time available to spend on instructional leadership and better work-life balance are among the top factors cited by administrators as reasons to stay in their profession [6]. Administrators are often on the “front lines” of school safety and policy implementation and are the focal point for accountability concerning school academic performance [10]. Thus, school administrators are in a unique position in which they are responsible for implementing and accountable for mandated policies and procedures but may lack needed support. Nevertheless, failed mandated policies and procedures can result in considerable stress and job dissatisfaction [12]. In addition, administrators report that high workload, lack of time for teaching and learning, and student and staff mental health issues are among the highest contributors of stress in their work [13]. For example, the nature of work in schools for administrators involves resolving disputes from multiple sources including parents and teachers. In a study of Israeli elementary and secondary school principals, it was found that when participants felt their leadership was being challenged by teachers and parents simultaneously, they were more likely to report feeling burned out [14]. Relatedly, working as a school administrator can be very isolating and prone to loneliness. In a series of qualitative interviews with school administrators in Istanbul, Yuksel and Ozgenel [15] found that participants felt like they did not have time for their families and were forced to cope with job-related stress on their own. Given such demands, research is needed to better understand administrator turnover and how school administrators’ wellbeing can be supported.

Indeed, research has documented the psychological benefits of emotional and professional support [16]. We also know that lack of administrator support has detrimental effects on teachers and can exacerbate the effects of violence [17]. While few studies have examined how district-level support contributes to retention intentions among school administrators, a recent longitudinal study of Australian and Irish principals demonstrated that support from supervisors positively impacts principal wellbeing over time [18]. Existing research also suggests support for principals varies in quality and consistency while often lacking structure and clarity [19]. To address this gap in the literature, in the present study, we assess the association between administrators’ perceptions of district support and their intentions to transfer/quit.

### 1.2. Administrator Wellbeing

School administrator wellbeing is an important factor to consider in understanding turnover, but it has received limited empirical investigation [20,21]. There are, however, studies that demonstrate the extent of administrators’ struggles with mental health, including recent work which estimates that nearly three-quarters of school administrators required greater emotional and mental health support [8], as evidenced by reported high rates of stress, burnout, and depression [22]. In this study, and drawing from the extant literature, we conceptualize wellbeing as a multifaceted construct that includes dimensions such as mental (anxiety/stress) and physical (safety) wellbeing [23,24].

Conceptually, district-level support (or lack of support) can have implications for administrators’ wellbeing and health [18]. In turn, administrators’ decisions to transfer schools or quit the profession can reflect appraisals concerning work demands, and accordingly, a coping response [6]. Thus, wellbeing may serve as an intermediary mechanism. Importantly, administrators’ perceptions of their safety may overlap with their experiences of violence and other safety concerns, or they may be vicarious based on the experiences of others in their school [25]. Nonetheless, perceptions of safety among administrators represent the overall dynamics of school climate and can be related to their global sense of wellbeing at work.

Research has found that when teachers feel supported by their administrators, they are more satisfied with their jobs [16,17] and feel safer and less stressed and anxious [25]. As a result, it is plausible that lower levels of stress and anxiety may then minimize transfer and quit intentions for teachers or administrators [26]. Studies have also documented that support from district offices can have myriad benefits for school administrators, including helping them carry out the responsibilities of their jobs and enhance their professional identities [19]. Conversely, lack of support can result in administrators struggling with the requirements of their jobs [27], ultimately contributing to higher levels of stress and desire to either transfer or quit [28]. Taken together, the research on administrator anxiety, stress and safety concerns at work suggests that these conditions may serve as mechanisms through which lack of district support may result in administrators’ decisions to leave their school or the profession altogether [20]. The present study aims to address this important area by examining administrators’ reported anxiety, stress and workplace safety as possible mediators of attrition decisions (transfer or quit).

While administrator reports of overall school safety are likely related to school violence [6], little is known about how administrators’ sense of safety is impacted by experiences of verbal threats and physical assaults, largely because administrator victimization is such a novel topic in the scientific literature. What is known points to an actionable link between administrators’ risk of workplace violence and the practices they implement [29]. For example, teachers who are at greater risk of victimization are more apt to utilize a range of school safety practices, such as mentoring, counseling, parent engagement, teacher training, community involvement, and physical security strategies (i.e., school hardening strategies such as police officers and metal detectors). Importantly, in Pyo’s study [29], the more teachers perceived a risk of higher workplace violence, the more likely they were to use physical security strategies compared to other methods. This difference is noteworthy, as the use of such strategies is not supported by research literature on school violence [30,31].

### 1.3. Administrator Victimization

Finally, a growing body of research literature has documented the problem of violence directed against PreK-12th grade educators and staff [32,33] and its adverse consequences. A recent national study indicated that the prevalence of student violence against administrators was 65% for verbal/threatening behaviors (e.g., obscene remarks or gestures, verbal harassment) and 42% for physically violent behaviors (e.g., objects thrown at them, physical attacks) before COVID in 2019–2020 [33]. Preliminary research demonstrates that administrators face violence from varied aggressors, including students, parents, and colleagues [13,33,34]. The most common aggressors are students, and as such, victimization from students was the focus of the current study.

Violence against administrators may be particularly exacerbated in regard to COVID-19 and identity-based learning, given the ensuing parental and community backlash many administrators experienced as a result of their policies [35]. Indeed, in one study, nearly one-third of principals reported experiencing threats from parents and community members as a result of their COVID-19 policies [6]. This problem extends beyond the U.S., with half of Australian principals reporting threats of violence and 38% reporting physical violence between 2011 and 2019 [34]. However, there have been few studies on the dynamics and outcomes of administrator-directed violence. Considering the prevalence of violence directed against administrators, it is important to consider administrator wellbeing and turnover intentions.

### 1.4. The Current Study

The goal of this investigation is to better understand the antecedents, mechanisms, and moderators of administrators’ desire to transfer schools or quit the profession. The current study addresses research gaps in the literature by examining the association between the direct and indirect influence of school administrators’ perceived district support on their transfer and quit intentions, and the mediating role of administrator safety and anxiety/stress. We also examined the role of violence directed against administrators by students as a moderator of these relations. We hypothesized the following:

(1) Higher levels of support from district leaders to school administrators will be negatively associated with their transfer and quit intentions;
(2) Administrator wellbeing (i.e., perceived school safety, anxiety/stress) will mediate the relations between perceived district support and their transfer and quit intentions;
(3) Violence against administrators will moderate the relations between support from district leaders, perceived safety, anxiety/stress, and transfer and quit intentions.

## 2. Materials and Methods

### 2.1. Participants

Participants included 457 PreK–12th grade administrators and principals from the United States who participated in a larger national study examining violence directed against educators and school personnel. Most respondents self-identified as assistant or vice principals (*n* = 198; 43.3%) and principals or heads of school (*n* = 196; 42.9%), followed by deans of students (*n* = 28; 6.1%), other administrative roles (*n* = 18; 3.9%), building administrators (*n* = 13; 2.8%), and deans of faculty (*n* = 2; 0.4%), with two respondents declining to provide their specific administrative roles (0.4%). Most participants identified as female (*n* = 261; 57.1%), followed by male (*n* = 195; 42.7%), with one respondent declining to provide an answer (0.2%). Most participants self-identified as Caucasian/White (*n* = 344; 75.3%), followed by African American/Black (*n* = 54; 11.8%), Hispanic/Latinx (*n* = 32; 7.0%), Multiracial (*n* = 14; 3.1%), Native American or Alaska Native (*n* = 4; 0.9%), other race (*n* = 3; 0.7%), and Asian-American/Asian (*n* = 2; 0.4%). Four participants did not provide information on race/ethnicity (0.9%).

Most participants worked in elementary schools (*n* = 145; 31.7%) and high schools (*n* = 145; 31.7%), followed by middle schools (*n* = 89; 19.5%) and PreK-12th grade schools (*n* = 24; 5.3%). Seventeen participants did not report their respective school level (3.5%). The majority of respondents reported working in suburban community settings (*n* = 200; 43.8%), followed by rural (*n* = 127; 27.8%) and urban (*n* = 128; 28.0%) settings. Two respondents did not provide the urbanicity of their schools (0.4%). Participants were 49 years old, on average (*SD* = 8.62), with an average of 9.26 years (*SD* = 7.37) of experience working in education.

### 2.2. Measures

A web-based survey was designed and disseminated by the APA Task Force on Violence Against Educators and School Personnel, representing scholars from universities and organizations across the United States. This APA Task Force collaborated with several national organizations during survey development and participant recruitment, including the National Education Association (NEA), American Federation of Teachers (AFT), National Association of School Psychologists (NASP), National Association of School Social Workers (NASW), and School Social Work Association of America (SSWAA). Participants completed demographic information and specific study measures via the online survey. The survey took about 25 min to complete. The survey was completed during the COVID-19 lockdown (2020–2021). When possible, confirmatory factor analysis (CFA) was used to affirm the psychometric properties of the scales, with commonly used model fit indices, such as root mean square error of approximation (RMSEA), comparative fit index (CFI), and standardized root mean square residual (SRMR) [36] to determine goodness of fit to the data.

#### 2.2.1. Perceived District Support of School Administrators

Items from the *Principal Transformational Leadership Survey* [37] were adapted to measure school administrators’ perceptions of their district leaders’ support of them. Three items asked administrators to rate, on a 5-point frequency scale of 0 (*Never*) to 4 (*Almost always*), how often they were supported by their district leaders (α = 0.89, ω = 0.89; e.g., “My district leadership supports me in matters of discipline”). Since the scale included only three items, CFA could not be used to affirm the psychometric properties of the scale, as a fully identified/saturated model occurred. This scale was validated as part of the full measurement model (see below).

#### 2.2.2. Administrator Perceptions of Safety

Administrators’ perceptions of safety during the 2019–2020 school year were measured via five items from the *Teachers’ Reactions to Violence Scale* [38]. Administrators were asked to rate on a 5-point frequency scale of 0 (*Not at all*) to 4 (*Almost always*) how often they felt safe at school (α = 0.79, ω = 0.82; “I felt safe when I came to school”). Higher scores, based on the latent factor structure, represented greater perceptions of safety. CFA affirmed the psychometric properties of the scale, which fit the data well (RMSEA = 0.02, CFI = 0.99, SRMR = 0.01).

#### 2.2.3. Administrator Anxiety/Stress

Administrators’ feelings of anxiety and stress were measured via three items [33]. Participants rated on a 5-point frequency scale ranging from 0 (*Not at all*) to 4 (*Almost always*) how often they experienced anxiety and stress during the 2019–2020 school year (α = 0.80, ω = 0.83; e.g., “I have anxiety when thinking about school”, “I find my work stressful”). Higher composite scores, based on latent scores, represented more anxiety and stress. As with the perceived district support scale, the anxiety/stress scale only included three items and therefore could not be verified with CFA. This scale was also validated in the larger measurement model (see below).

#### 2.2.4. Administrator Transfer/Quit Intentions

Administrators’ intentions to transfer schools or quit their role as a school administrator were measured via two subscales, each of which consisted of three items [33]. Administrators rated on a 5-point Likert-type scale, from 1 (*Strongly disagree)* to 5 (*Strongly agree*), their intentions to transfer schools (α = 0.89, ω = 0.89; e.g., “I plan to transfer to a different position or school/district”) or quit the profession (α = 0.89, ω = 0.90; e.g., “I plan to quit my profession or retire early”). Higher composite scores, based on the latent factor structure, represented greater intentions to either transfer or quit. CFA affirmed the psychometric properties of the scale, which fit the data well (RMSEA = 0.14, CFI = 0.97, SRMR = 0.05), albeit with an elevated RMSEA value. This was ignored, as an inflated RMSEA value in an otherwise excellent-fitting model is not justification for model rejection [39].

#### 2.2.5. Administrator-Directed Violence

The *Educator Victimization Scale* [33] was used to examine administrator-directed violence from students. Administrators rated on a frequency scale, from 0 (*Never*) to 5 (*Daily*), how often they experienced 15 different types of victimization from students during the 2019–2020 school year (Pre-COVID, August, 2019 to March, 2020; α = 0.89, ω = 0.89; “I was physically attacked (e.g., bitten, scratched, hit)”; “I was verbally threatened”). Higher composite scores, based on the latent factor, represented greater reports of victimization. CFA again affirmed the psychometric properties of the measure (RMSEA = 0.15, CFI = 0.91, SRMR = 0.06), and we again ignored the slightly elevated RMSEA value [39].

### 2.3. Procedure

Following Institutional Review Board (IRB) procedures approved by the University of North Carolina at Chapel Hill, online survey data were collected from August 2020 to June 2021. School participants were contacted via school emails provided by a national marketing firm (MCH Strategic Data) and/or in conjunction with national partners that posted on social media or sent emails to a sample of their constituents. MCH gathers teacher contact information by conducting website scans of public education data sources and importing this information into a comprehensive database of 5.4 million school staff nationwide. This information is continuously verified to ensure contact and school information are current. MCH periodically contacts individuals within this database to allow them to opt-out of the list. Participants were provided a link to the online survey describing the study’s purpose, and survey completion indicated assent. Participant data used in this study were de-identified.

### 2.4. Data Analytic Plan

Using Mplus Version 8, structural equation modeling (SEM) was conducted with perceptions of district support predicting intentions to transfer schools and/or quit school administration both directly and indirectly through safety and anxiety/stress. Further, victimization from students served as a moderator of these relations. Administrators’ demographic characteristics, including their gender, race/ethnicity, school level, age, and school urbanicity (i.e., rural, suburban, or urban), were included in all analyses as covariates. The model was analyzed first without interaction effects to ascertain the main effects of study variables. Model constraint commands in Mplus were used to compute indirect effects, which were examined via 95% confidence intervals generated from 1000 bootstrapped samples. The same model was then analyzed a second time with the interaction effects of school administrator victimization included. The XWITH command in Mplus was utilized to compute the interaction between school administrator victimization and perceived district-level support. R version 4.3.0 was used to create an interaction plot by grouping participants into low and high victimization based on ± 1 standard deviation away from the average reported victimization. Full information maximum likelihood estimation was utilized for all analyses to handle missing data.

## 3. Results

### 3.1. Preliminary Analyses

Descriptive statistics and correlations among study variables are presented in Table 1. Correlations were all statistically significant with interpretable effect sizes [40] and in the expected directions. Prior to running the primary analyses, we tested a measurement model to affirm the psychometric properties of the scales when modeled in tandem. The previously validated factor structures for all scales were included in the measurement model, which exhibited excellent fit to the data (RMSEA = 0.05, CFI = 0.96, SRMR = 0.05). Thus, we proceeded with primary analyses using the selected measures.

### 3.2. Relations Between District Support, Safety, Anxiety/Stress, and Attrition Intentions

**Figure 1:**
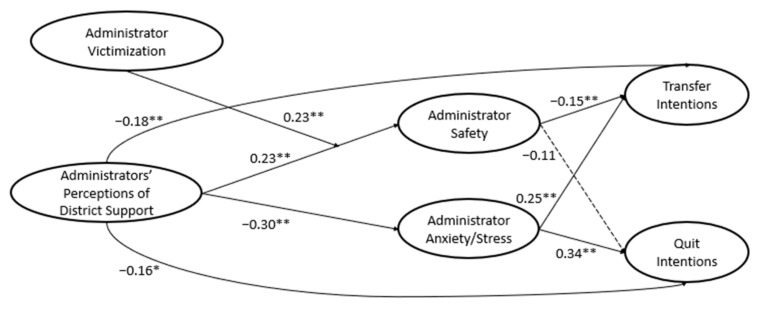
SEM path analysis findings. *Note. N* = 457. * *p* < 0.05. ** *p* < 0.01. All reported coefficients are standardized estimates. Solid lines coincide with statistically significant effects and dotted lines coincide with nonsignificant effects. Covariates (i.e., gender, race/ethnicity, school level, age, and school urbanicity) were not included for figure simplicity. *R*^2^ was 0.19 for transfer intentions and 0.21 for quit intentions.

Significant main effects and interactions—all standardized coefficients—are presented in Figure 1 along with the R^2^ values for both transfer and quit intentions. Perceptions of district-level support by administrators were directly and negatively related to both transfer and quit intentions. In other words, the more administrators believed that their district leaders supported them in matters of discipline, the less likely they were to transfer schools/districts or to quit the profession, supporting our first hypothesis. Further, district support was positively related to administrators’ sense of safety and negatively related to their anxiety/stress. Additionally, administrators’ sense of safety negatively related to transfer intentions and anxiety/stress was positively related to both transfer and quit intentions. Administrators’ sense of safety was not significantly related to quit intentions.

### 3.3. Indirect Effects Between District Support and Attrition Intentions

The indirect effects are presented in Table 2 as standardized coefficients. The indirect effects of district support on transfer and quit intentions yielded mixed results. Anxiety/stress emerged as a significant mediator—the indirect effects of district support on transfer and quit intentions through anxiety/stress were both statistically significant. District support for administrators was negatively related to anxiety/stress, which in turn was positively related to transfer and quit intentions. Conversely, neither the indirect effect for district support through sense of safety for transfer intentions nor quit intentions was statistically significant. In other words, perceptions of safety did not mediate the relations between district support and transfer and quit intentions, so our second hypothesis was partially supported.

### 3.4. Moderation of Administrator-Directed Violence

**Figure 2:**
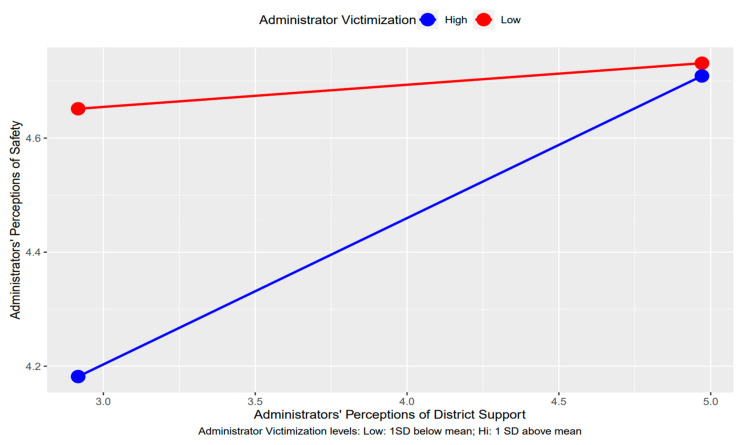
Interaction of administrators’ perceptions of district support and victimization on safety. *Note.* High and low groupings of victimization were based on ± one SD above and below the mean.

Administrator-directed violence from students moderated the relation between district support and safety (Figure 1). An examination of an interaction plot (Figure 2) revealed that the positive relation between district support and safety was stronger among frequently victimized administrators, compared to those who reported low levels of victimization. For those reporting low victimization, perceptions of safety remained relatively high regardless of district-level support. No other relationship was significantly moderated by victimization, so there was partial support for our third hypothesis.

## 4. Discussion

This study was among the first to examine antecedents to administrator turnover in the context of district support, wellbeing, and administrator-directed violence. Our findings indicate that school administrators’ experiences of district level support have a direct and negative influence on their intentions to transfer schools or quit their jobs. These findings are encouraging—the more administrators feel supported, the less likely they are to leave their positions. This finding is consistent with previous research showing the power of social support for school administrator effectiveness and retention [18,41].

### 4.1. Indirect Effects of District Support on Retention Through Wellbeing

We found that administrators’ anxiety/stress mediated the relations between district support and transfer/quit intentions. When administrators feel less anxious and stressed because of the support they receive from their district leaders, they in turn are more likely to remain in their positions. Results from the current study are supported by a recent study of nearly 3000 Australian and Irish principals, which longitudinally demonstrated the connection between support from one’s superiors and improved mental health [18]. Similarly, these findings resonate with other studies that have found increased likelihood of burnout among administrators when they are stressed [26] and that support structures (e.g., professional development, coaching, etc.) are useful in improving administrators’ job satisfaction, which is essential to administrator retention [27]. Likewise, anxiety and stress have been linked to both victimization and intentions to transfer or quit among teachers [42]. Our findings highlight the need for targeted professional development and support across the school system hierarchies and in particular for school leaders, which is an under-studied area. Indeed, district leaders who provide sustained support for their administrators can help bolster mental health and wellness and possible administrator retention.

Contrary to our hypotheses, administrators’ sense of safety did not mediate the relationship between district support and transfer and quit intentions. It may be possible that school administrators’ sense of school safety may not be related to the more general support they receive from their district supervisors. District support officials may be too distal from the school environment to affect school administrators’ sense of personal safety. However, it should be noted that, even though administrators’ safety did not operate as a mechanism through which district support related to transfer or quit intentions, it does play a role for administrators who experience higher violence given the significant moderation found in our study (see Figure 2). Further, workplace safety is important and negatively associated with transfer and quit intentions directly [25]. Future research is warranted to further examine these important nuances.

### 4.2. Administrator Victimization as Moderator

The current investigation found violence from students against administrators moderated the relation between administrators’ experiences with district support and safety. Administrators who reported low levels of victimization experienced high perceptions of safety at all levels of district support. For those who experienced high levels of victimization, higher district support was strongly and positively associated with higher perceptions of safety. This finding highlights the importance and benefit of district support for administrators who have experienced victimization. While not specifically related to violence, administrators have reported positive experiences with tangible support from district staff, including tools such as rubrics, self-evaluations, and “buffering” strategies, which protect principals from extraneous work demands that prevent them from engaging in other important activities [19]. Support from district staff may be particularly important given the general lack of mental health and stress-reduction resources tailored to the unique experiences of administrators [43]. This finding should serve as a call to action for researchers, school practitioners and policy makers to ensure consistent access to practical support for school administrators, especially those serving in schools most susceptible to violence. Policymakers and other school stakeholders (parents, students, staff, educators) should make every effort to reduce administrator victimization while also fostering healthy relationships between administrators, students, educators and district leadership, all in pursuit of increased leadership retention, positive school and work environments, and ultimately better learning environments for students.

### 4.3. Limitations

This study has limitations. First, this study included self-report measures from school administrators. Though self-report measures have been demonstrated to be effective for survey research, self-report tools may include biases and the potential for inaccurate responses, warranting empirical investigation. Additionally, our measure of district support was not specific to violence directed at administrators from students, but rather about support in matters of discipline generally. Next, the data used in this study were cross-sectional, precluding the possibility to generate causal inferences. Finally, though this study employs a large sample from respondents across the United States, it is inherently a convenience sample; those who chose to respond to the survey may have had stronger opinions about their district-level support, wellbeing, and victimization than other administrators. Annual surveys across school stakeholders would contribute to a comprehensive understanding of safety, mental health, and retention issues, as well as targeted recommendations for training, support, and school improvement.

### 4.4. Implications for Research and Practice

There are few studies examining administrator-directed violence and turnover, and our findings indicate significant rates of victimization and intentions to leave their positions. More research is needed that explores administrator experiences, causes, correlates, and protective factors across the school ecology and across school contexts. Longitudinal and mixed-methods research is needed to understand and address school leader needs. Qualitative research (e.g., interviews and focus groups) including multiple sources of data (e.g., observational data, human resource records) would be useful, given limited data on this population. Further, many measures of wellbeing do not address administrators’ lived experiences or the specific aspects of wellbeing we hope to address [44]. We also need to carefully examine career decision-making and actual quit and transfer rates.

Our findings underscore the critical need to develop and validate interventions and support systems for promoting school administrators’ safety, wellbeing and retention. School administrators are ultimately responsible for school performance accountability and the safety of teachers, staff, and students. Thus, many administrators can be alone as they face work-related challenges that compound their stress, anxiety, and victimization. Importantly, we found that district leadership support was meaningful for school administrators’ mental health, safety, and retention, which may serve as a focal area to leverage in future interventions. Considering that schools experience disparate rates of violence, even within school districts, district leaders should identify schools with elevated rates of violence and develop targeted strategies to support and provide training to principals in these settings. Such strategies should consider school-wide interventions, supports, and professional interpersonal connections between district and school-level administrators—principals need to feel supported as professionals and school leaders. Thus, district leadership should offer proactive, sustained systems of support for school administrators that are school-wide, culturally sensitive, context-dependent, and tailored to their unique needs.

## 5. Conclusions

It is crucial that district leaders provide their school administrators emotional and professional support. District support serves as a powerful factor to improve school administrators’ perceived workplace safety and reduce their feelings of anxiety/stress, but it also directly and indirectly relates to their intentions to remain in or leave their schools. Improving administrator retention is critically important for enhancing and sustaining the scholastic and mental health outcomes of teachers and students in schools. Finally, every effort should be made to minimize or eliminate violence perpetrated against administrators, as this directly affects the degree to which district support is effective in making administrators feel safer and is likely a contributing factor to administrator turnover.

## Figures and Tables

**Table 1:** Descriptive statistics and correlations of school administrator-reported scales.

	*M*	*SD*	1	2	3	4	5	6
1. District-level support	3.97	1.00	-					
2. School safety	4.54	0.68	0.27 **	-				
3. Anxiety/stress	2.45	0.79	−0.32 **	−0.26 **	-			
4 Transfer intentions	1.53	0.91	−0.32 **	−0.26 **	0.32 **	-		
5. Quit intentions	1.89	1.12	−0.26 **	−0.23 **	0.35 **	0.54 **	-	
6 Administrator victimization	1.82	0.99	−0.24 **	−0.29 **	0.30 **	0.21 **	0.13 *	-

Note. *n* = 457. * *p* < 0.05. ** *p* < 0.01. Covariates were not included for table simplicity.

**Table 2:** Indirect effects between district support and retention through safety and anxiety/stress.

Indirect Effects (Mediations)	*β*	*S.E*	*p*	95% CI [*LLCI*, *ULCI*]
Support → Safety → Intentions to transfer	−0.03	0.02	0.08	[−0.06, −0.00]
Support → Safety → Intentions to quit	−0.03	0.02	0.12	[−0.05, 0.00]
Support → Anxiety/stress → Intentions to transfer	−0.06	0.03	0.01	[−0.10, −0.02]
Support → Anxiety/stress → Intentions to quit	−0.10	0.03	<0.01	[−0.16, −0.05]

*Note. N* = 457. Confidence intervals are reported with a bootstrap sample size = 1000. *LLCI* = Lower level of the 95% bootstrap confidence interval; *ULCI* = Upper level of the 95% bootstrap confidence interval. The lower level of the indirect effect of support on intentions to transfer was technically below zero (−0.002), but the *p*-value was above 0.05 and the value was so close to zero that we chose to interpret that finding as not statistically significant.

## Data Availability

The data are not currently available for public access due to the size of the data sets, which are currently undergoing ongoing data cleaning, organizing, scale refinement, and scale validation. The study materials are available upon request.
